# A Spectrally Compatible Pseudo-Panchromatic Intensity Reconstruction for PCA-Based UAS RGB–Multispectral Image Fusion

**DOI:** 10.3390/jimaging12030122

**Published:** 2026-03-11

**Authors:** Dimitris Kaimaris

**Affiliations:** School of Spatial Planning and Development, Aristotle University of Thessaloniki, 54124 Thessaloniki, Greece; kaimaris@auth.gr

**Keywords:** aerial remote sensing, UAS, RGB, multispectral sensor, pseudo-panchromatic image, image fusion, band-wise correlation, spectral angle mapper, Tenengrad, Laplacian variance

## Abstract

The paper presents a method for generating a pseudo-panchromatic (PPAN) orthophotomosaic that is spectrally compatible with the multispectral (MS) orthophotomosaic, and it targets the fusion of unmanned aircraft system (UAS) RGB–MS orthophotomosaics when no true panchromatic band is available. In typical UAS imaging systems, RGB and multispectral sensors operate independently and exhibit different spectral responses and spatial resolutions, making the construction of a spectrally compatible substitution intensity a critical challenge for component substitution fusion. The conventional RGB-derived PPAN preserves spatial detail but is constrained by RGB–MS spectral incompatibility, expressed as reduced corresponding-band similarity. The proposed hybrid intensity (PPAN_E_) increases the mean corresponding-band correlation from 0.842 (PPAN_A_) to 0.928 (PPAN_E_) and reduces the across-site mean SAM from 5.782° to 4.264°, while maintaining spatial sharpness comparable to the RGB-derived intensity. It is proposed that the PPAN_E_ orthophotomosaic be produced as a hybrid intensity (single band) image. Specifically, a multispectral-visible-derived intensity is resampled onto the RGB grid and statistically integrated with RGB spatial detail, followed by mild high-frequency enhancement to produce the final PPAN_E_ orthophotomosaic. Principal Component Analysis (PCA) fusion is applied to seven archaeological sites in Northern Greece. Spectral quality is evaluated on the MS grid using band-wise (corresponding-band) correlation and the Spectral Angle Mapper (SAM), while the spatial sharpness of the fused NIR orthophotomosaic is assessed using Tenengrad and Laplacian variance. The PPAN_E_ orthophotomosaic consistently increases correlations relative to PPAN_A_ (especially in Red Edge/NIR) and reduces the mean site-mean SAM. PPAN_C_ yields the lowest SAM but also the lowest spatial sharpness/clarity, whereas PPAN_E_ maintains spatial sharpness/clarity comparable to PPAN_A_, supporting a balance between spectral consistency and spatial detail, as also confirmed through comparative evaluation against established component substitution fusion methods. The approach is reproducible and avoids full histogram matching; instead, it relies on explicitly defined linear standardization steps (mean–std normalization) and controlled spatial sharpening, and performs consistently across different scenes.

## 1. Introduction

Image fusion has been studied extensively for satellite panchromatic (PAN) and multispectral (MS) data [1,2,3,4,5,6,7,8,9]. In the case of image fusion using Unmanned Aerial System (UAS) data, most of the sensors in use unfortunately do not acquire PAN imagery, because they do not include dedicated PAN sensors (with a few exceptions of commercial systems [10,11,12] that capture PAN and MS images simultaneously). Consequently, in the majority of UAS applications it is not possible to obtain PAN images, in contrast to most satellite applications where PAN and MS images are collected simultaneously and used together. In addition, the RGB and MS sensors used on UAS platforms have different spectral characteristics, and their RGB imagery typically has much higher spatial resolution than the MS imagery.

Existing pansharpening methodologies can be broadly categorized into Component Substitution (CS), Multi-Resolution Analysis (MRA), and hybrid or model-based approaches. CS techniques, including Principal Component Analysis (PCA), Intensity–Hue–Saturation (IHS), Gram–Schmidt and Brovey transforms, inject spatial detail through intensity substitution but may introduce spectral distortion when spectral compatibility between the substituted intensity and the multispectral dataset is limited [1,6,7,8,9]. This distortion arises because the substituted intensity component is assumed to approximate the spectral behaviour of the multispectral bands, an assumption that is often violated when the intensity originates from a different sensor with broader or mismatched spectral responses [3]. Several studies have therefore investigated strategies to improve spectral consistency in component substitution frameworks, including adaptive intensity construction, histogram matching, and sensor-response-based modelling [6,8,9]. However, most of these approaches assume the availability of a native panchromatic band or operate within a single-sensor imaging system where spectral response compatibility is implicitly satisfied [1,6,7]. In contrast, UAS RGB–multispectral fusion represents a distinct scenario in which the substituted intensity must be reconstructed from a different sensor with different spectral responses and spatial characteristics. In such cross-sensor conditions, the intensity component becomes the primary source of spectral distortion in PCA-based substitution. MRA approaches, such as wavelet or Laplacian pyramid methods, transfer spatial frequencies more conservatively but still rely on the availability of a spectrally compatible high-resolution band [6,7,8,9].

Nevertheless, since 2020 a systematic research effort [13,14,15,16] has been underway across several archaeological areas (seven in total) to produce fused imagery from the derived UAS RGB and MS orthophotomosaics. The aim is for the fused image to retain the high spatial resolution of the RGB orthophotomosaic while exhibiting strong spectral correlation with the original MS orthophotomosaic. For georeferencing the RGB and MS images, either PPK (Post-Processed Kinematic) processing or ground control points (GCPs) are employed. Digital surface models (DSMs), as well as RGB and MS orthophotomosaics, are produced with full spatial co-registration (in the same reference system). This is critical, because even small mis-registrations between products can substantially degrade the spectral outcome of the fused image [1]. Subsequently, following the methodological workflow, a pseudo-panchromatic (PPAN) image is generated from the RGB orthophotomosaic, and image fusion is performed via PCA using all bands of the MS orthophotomosaic, retaining all components, replacing the first principal component (PC1) with the PPAN, and applying the inverse transformation to generate the fused image. Although ideally the correlation between the original spectral information and that of the fused image should exceed 0.9 [17,18], analysis of the correlation matrices from experiments conducted to date indicates that the workflow enables satisfactory (0.8 to 0.9) preservation of the relative (correlation-based) spectral behaviour of the MS data in the fused image. This is mainly attributed to the lack of spectral compatibility between the PPAN and the MS orthophotomosaic, which is associated with spectral distortion in the fusion result.

Despite the extensive use of component substitution fusion, limited attention has been given to the problem of intensity reconstruction under cross-sensor conditions, where no true panchromatic band exists and the substitute intensity is spectrally incompatible with the multispectral dataset. This gap becomes particularly relevant in UAS applications where RGB and multispectral sensors are operated independently and no dedicated panchromatic channel is available. In these conditions, the substituted intensity must simultaneously satisfy two competing requirements: spectral compatibility with the multispectral dataset and spatial detail consistent with the RGB imagery. The present study addresses this specific cross-sensor intensity reconstruction problem. The principal contribution of this work is a reproducible single-band intensity reconstruction (PPAN_E_) designed specifically to address RGB–MS spectral incompatibility in PCA-based fusion. Rather than introducing a new individual fusion operator, the proposed method formulates a compatibility-constrained intensity reconstruction strategy for cross-sensor substitution scenarios where no native panchromatic band exists. The reconstruction is formulated as a statistically constrained hybrid intensity model, in which MS-visible spectral affinity and RGB spatial gradients are integrated within a normalized intensity space prior to controlled high-frequency injection. In contrast to conventional RGB-derived intensity substitution, which directly injects spatial detail without considering cross-sensor spectral compatibility, the proposed reconstruction establishes improved spectral compatibility with the MS dataset while retaining high spatial sharpness. The contribution does not modify the underlying PCA substitution transform but introduces a compatibility-constrained intensity reconstruction formulated to mitigate cross-sensor spectral distortion prior to substitution. The reconstruction pipeline (PPAN_A_ → PPAN_C_ → PPAN_D_ → PPAN_E_) also provides a structured progression that allows the contribution of each processing stage to be evaluated explicitly within the same experimental framework A dedicated MS-derived control intensity (PPAN_C_) is additionally introduced to bound the spectral–spatial trade-off under a consistent evaluation framework across seven archaeological sites. In addition, a control experiment is adopted in which the intensity is exclusively the MS-visible-derived intensity (resampled onto the RGB grid without introducing new spatial information), in order to document the trade-off between maximum spectral affinity with the original MS orthophotomosaic and spatial sharpness. Also, evaluation of the fused products is performed comparatively using band-wise correlation and the Spectral Angle Mapper (SAM) index [19,20,21], while spatial sharpness is assessed in the NIR using Tenengrad [22,23] and Laplacian variance [23,24,25,26,27,28]. Finally, to further position the proposed methodology within the broader pansharpening literature, comparative fusion experiments are also conducted using established component substitution techniques under a common evaluation framework. In this paper, spectral preservation/transfer refers to preserving relative (correlation-based) spectral behaviour and to reducing spectral distortion as captured by SAM. This evaluation focuses on relative spectral consistency, expressed through band co-variation and spectral-angle distortion, rather than on absolute radiometric accuracy. Potential band-wise radiometric bias, such as mean reflectance offsets, is therefore not treated as a primary performance criterion within the present multi-sensor fusion context. The above processing steps were carried out in Erdas Imagine Professional 2023© v.16.8.1 (Hexagon AB, Stockholm, Sweden), and in some cases Microsoft Excel© worksheets were also used (Microsoft Corporation, Redmond, WA, USA).

**Figure 1:**
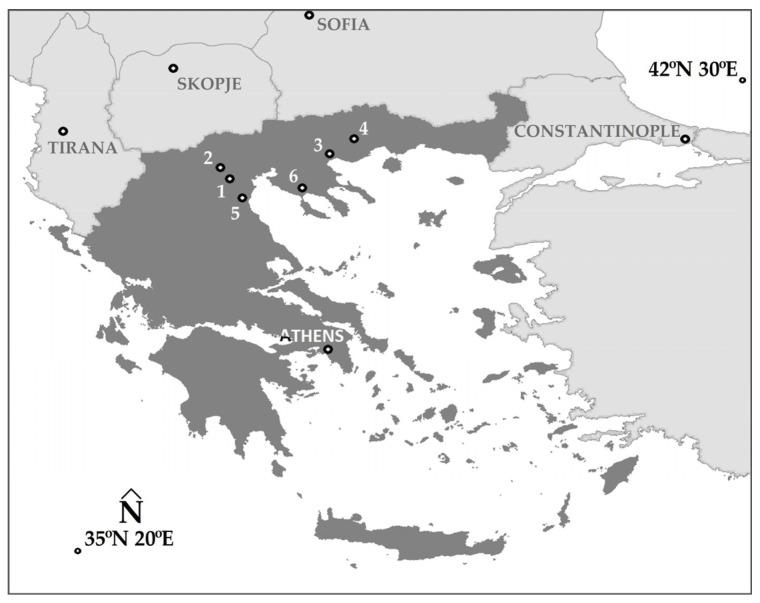
The archaeological sites where the methodology was applied: 1. Sanctuary of Eukleia; 2. Ancient Theatre of Mieza; 3. Kasta Tumulus and Early Christian Basilica C; 4. Acropolis of Platania; 5. Mosaic at Dion; 6. Olynthus.

The seven archaeological sites (all in Northern Greece, Figure 1) where the methodology was applied are the Sanctuary of Eukleia (4th century BC [29], 40°28′47.61″ N 22°19′16.87″ E) at the archaeological site of Aigai (Vergina), the Ancient Theatre of Mieza (2nd century BC [30], 40°38′38.6″ N 22°07′21.3″ E), the Kasta Tumulus (last quarter of the 4th century BC [31], 40°50′21.5″ N 23°51′44.9″ E), the Acropolis of Platania (from prehistoric times until late Roman antiquity [32], 41°11′05.4″ N 24°26′03.2″ E), the mosaic (~AD 200 [13], 40°10′33.53″ N 22°29′30.23″ E) at the archaeological site of Dion, the Early Christian Basilica C (5th–6th century AD [33], 40°49′14.04″ N 23°50′44.84″ E) at the archaeological site of Amphipolis, and the archaeological site of Olynthus (late 5th–early 4th century BC [34], 40°17′47.52″ N 23°21′15.37″ E).

The proposed PPAN_E_ formulation therefore addresses the specific problem of intensity reconstruction in cross-sensor RGB–multispectral fusion, providing a reproducible strategy to balance spectral compatibility with spatial detail prior to PCA substitution.

## 2. Methodology and Results

### 2.1. Data Preparation and Preprocessing

The workflow for producing DSMs and orthophotomosaics follows a standard UAS processing pipeline and is summarized below.

For image acquisition at five of the seven archaeological sites, the UAS WingtraOne GEN II (Wingtra AG, Zurich, Switzerland) [35] was used, equipped with an RGB sensor (Sony RX1R II (Sony Group Corporation, Tokyo, Japan), 35 mm focal length, 42.4 MP) and an MS sensor (MicaSense RedEdge-MX (MicaSense Inc., Seattle, WA, USA), 5.5 mm focal length, 1.2 MP; spectral bands: Blue, Green, Red, Red Edge, and Near-Infrared (NIR)) [35,36]. For image acquisition at two of the seven archaeological sites, the UAS DJI Phantom 4 (DJI, Shenzhen, China) [37] was used, equipped with an RGB sensor (integrated camera, 1/2.3″ CMOS, 12.4 MP) and an MS sensor (Sequoia+ (Parrot S.A., Paris, France), 3.98 mm focal length, 1.2 MP; spectral bands: Green, Red, Red Edge, and NIR) [38]. Because the two multispectral sensors used in this study (RedEdge-MX and Sequoia+) exhibit different spectral response functions and bandpass configurations, an additional cross-sensor consistency check was performed. Site-level spectral metrics (correlation and SAM) were stratified by sensor type to evaluate whether PPANE behaviour remains stable under varying spectral response characteristics. For image georeferencing, ground control points (GCPs) were surveyed, and for quality control of the final products (digital surface models/DSMs and orthophotomosaics), check points (CPs) were surveyed using a Topcon HiPer SR GNSS receiver (Topcon Positioning Systems, Tokyo, Japan) [39]. The same receiver was used in the WingtraOne GEN II deployments to measure the base point, enabling automatic georeferencing (Post-Processed Kinematic—PPK workflow) of the images in WingtraHub© v2.18.1 (Wingtra AG, Zurich, Switzerland). The RGB and MS orthophotomosaics were generated in a common reference system and with full spatial co-registration through a standard processing workflow in Agisoft Metashape Professional© v2.0.3 (Agisoft LLC, Saint Petersburg, Russia) [13,14,15,16], either using images already georeferenced via the PPK workflow or using GCPs (Table 1).

For the MS datasets at all archaeological sites, spectral/radiometric calibration was applied immediately after importing them into Agisoft Metashape Professional© v2.0.3 (Agisoft LLC, Saint Petersburg, Russia), using a calibration panel recorded before and after each flight. This converted the original digital numbers to per-band reflectance and reduced illumination-related variability. The reflectance values of the MS orthophotomosaics were used on the same scale produced by the calibration/export procedure (scaled reflectance); therefore, the values do not necessarily fall within the 0–1 range. RGB orthophotomosaics were used as DN-scaled sensor products (i.e., not as absolute reflectance). Further details of the processing workflow (e.g., flight planning, alignment, dense point clouds, DSM/orthophotomosaic parameters, and accuracy checks) are provided in the relevant publications [13,14,15,16].

### 2.2. Generation of PPAN_A_ from the RGB Orthophotomosaic

The first step, up to and including the present paper, was to convert the RGB orthophotomosaic to grayscale in Adobe Photoshop© CS6 v.13.0 (Adobe Inc., San Jose, CA, USA) (via Image/Mode/Grayscale) in order to produce the PPAN_A_ orthophotomosaic. This procedure was not based on a fixed linear combination of the RGB channels, but on a colour-managed conversion to the active grayscale working space; consequently, the output depends on the colour profiles in use. For all experiments presented in this paper, PPAN_A_ was generated using Adobe Photoshop grayscale conversion under fixed colour-management settings (sRGB IEC61966-2.1; Gray Gamma 2.2). Equation (1) is provided as the fully specified reproducible alternative for future applications. The grayscale conversions performed in Photoshop correspond functionally to a luminance-weighted linear transformation consistent with Equation (1). An equivalent transformation can therefore be implemented explicitly in programming environments (e.g., Python, MATLAB, GDAL) using linear band-weighting coefficients.

(1) $${PPAN}_{A}=(0.114\cdot Blue)+(0.587\cdot Green)+(0.299\cdot Red)$$

The luminance weighting in Equation (1) corresponds to the luma coefficients of the ITU-R BT.601 standard [40], which define the Y component as a linear combination of the Red, Green, and Blue bands. This particular weighting is used in the literature [41,42] in the same form for RGB image processing and luminance estimation. In those studies, the equation is employed to compute a luminance (Y) channel rather than to create a PPAN image. In the present paper, the same mathematical principle is used in a different context. The Blue, Green, and Red bands of the RGB orthophotomosaic are combined using these coefficients to produce the PPAN_A_ orthophotomosaic, which is spectrally consistent with the RGB orthophotomosaic because it originates from the same sensor and the same bands. Clearly, in neither case, whether using Adobe Photoshop© CS6 v.13.0 (Adobe Inc., San Jose, CA, USA) or Equation (1), is any spectral adjustment performed with respect to the bands of the MS orthophotomosaic. As a result, the PPAN_A_ orthophotomosaic remains a product of the RGB sensor, whose spectral response is not identical to the spectrally narrower bands of the MS sensor. In other words, the pixel values of the PPAN_A_ orthophotomosaic reflect the spectral response of the RGB sensor rather than that of the MS sensor.

**Figure 2:**
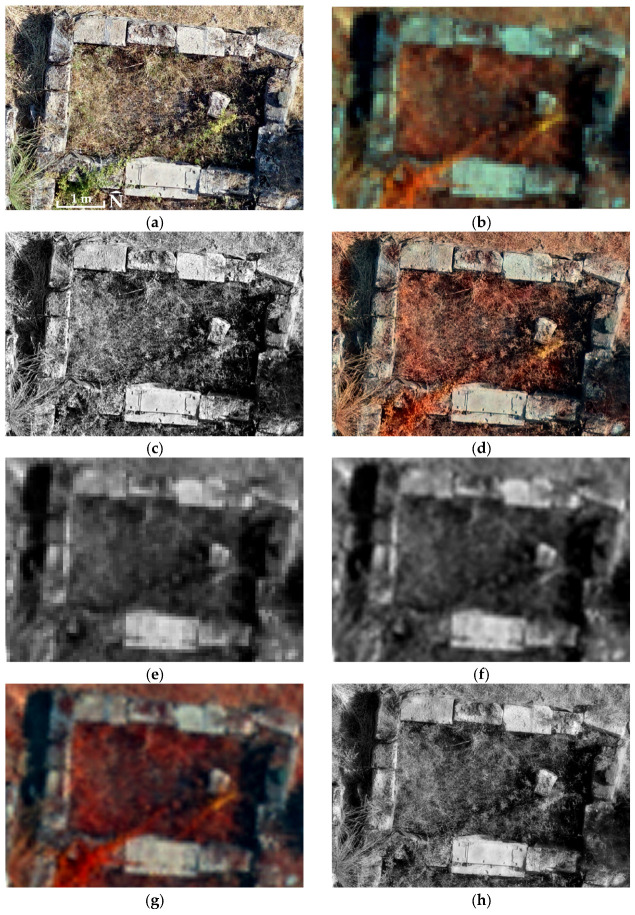

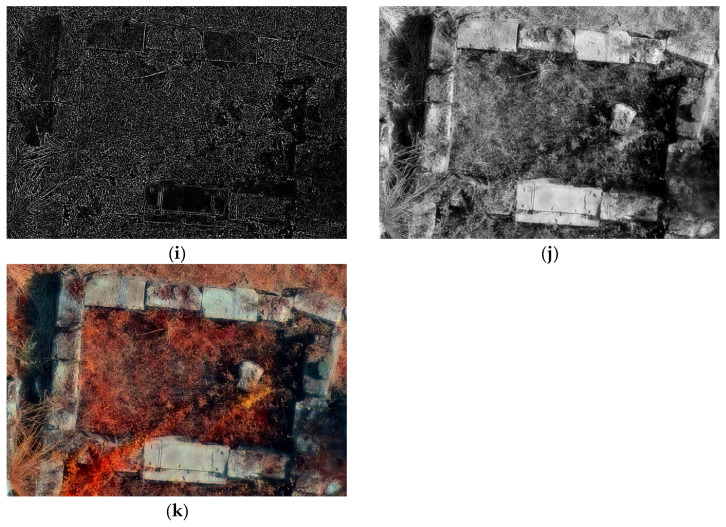
Orthophotomosaic excerpts from the Sanctuary of Eukleia: (**a**) RGB; (**b**) MS (Bands Blue, Green, NIR); (**c**) PPAN_A_; (**d**) Fused with PPAN_A_ (Bands Blue, Green, NIR); (**e**) PPAN_B_; (**f**) PPAN_C_; (**g**) Fused with PPAN_C_ (Bands Blue, Green, NIR); (**h**) PPAN_D_; (**i**) PPAN_D,HP_, (**j**) PPAN_E_, (**k**) Fused with PPAN_E_ (Bands Blue, Green, NIR). Centre of images 40°28′47.34″ N 22°19′17.76″ E.

**Figure 3:**
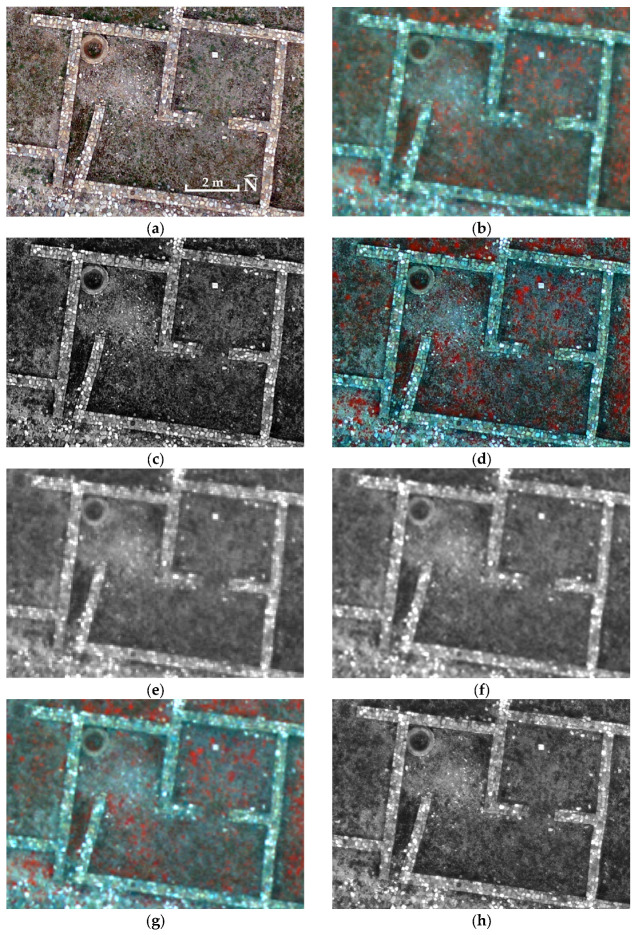

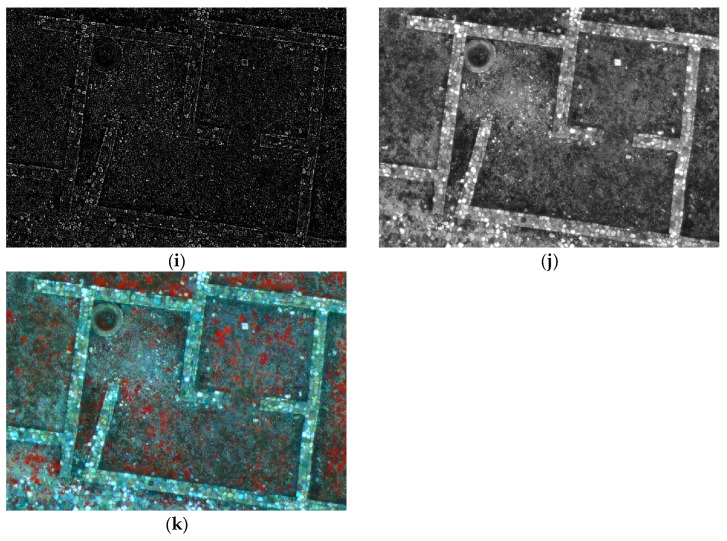
Orthophotomosaic excerpts from Olynthus: (**a**) RGB; (**b**) MS (Bands Blue, Green, NIR); (**c**) PPAN_A_; (**d**) Fused with PPAN_A_ (Bands Blue, Green, NIR); (**e**) PPAN_B_; (**f**) PPAN_C_; (**g**) Fused with PPAN_C_ (Bands Blue, Green, NIR); (**h**) PPAN_D_; (**i**) PPAN_D,HP_, (**j**) PPAN_E_, (**k**) Fused with PPAN_E_ (Bands Blue, Green, NIR). Centre of images 40°17′46.13″ N 23°21′16.97″ E.

Tests carried out across all experimental sites using Equation (1) to generate the PPAN_A_ orthophotomosaics (Figure 2 and Figure 3) yielded essentially the same band-to-band correlation matrices between the original MS orthophotomosaics and the fused images as those already computed in previous papers [13,14,15,16]. Therefore, the use of Equation (1) in new applications is not intended to improve the spectral quality of the PPAN_A_ orthophotomosaic or the final fused image, but to apply a fully specified transformation with explicit and known RGB combination weights, thereby improving the transparency and reproducibility of the workflow.

It is noted that the BT.601 coefficients represent standard luma weights and are not derived from the spectral response functions of the specific RGB sensor used in each flight. Their role here is to replace colour-managed grayscale conversion with an explicit, sensor-independent linear transform.

A more physically grounded alternative would involve deriving weighting coefficients directly from the measured spectral response functions of the RGB sensor and their overlap with the multispectral bandpasses. Such sensor-response-based weighting schemes could constitute a direction for future research in order to enhance physical spectral compatibility.

### 2.3. Generation of PPAN_B_ from the MS Orthophotomosaic

To ensure that the PPAN orthophotomosaic exhibits visible-band spectral responses similar to those of the MS sensor, Equation (1) is applied to the first three bands (Blue, Green, Red) of the MS orthophotomosaic to produce the PPAN_B_ orthophotomosaic (Figure 2 and Figure 3), i.e., an MS-visible-derived intensity image. In this way, spectral affinity is achieved between PPAN_B_ and the MS orthophotomosaic, in contrast to PPAN_A_, which is derived from the RGB orthophotomosaic. The coefficients of Equation (1) are used here as a practical and fully reproducible linear transform to construct an intensity image from the visible MS bands, rather than as a physical/photometric model of luminance for narrow-band spectral responses. The objective is for PPAN_B_ (and subsequently PPAN_C_) to represent an intensity image derived from, and consistent with, the visible bands of the MS orthophotomosaic (MS-visible-derived intensity), so that it can serve as a reference within the PCA substitution framework. As an additional test, an equal-weight (0.33–0.33–0.34)-intensity composition from the visible bands was also examined, without materially changing the conclusions regarding the relative ranking of the fused images produced using PPAN_A_, PPAN_C_, and PPAN_E_. A further distinction is that PPAN_A_ inherits the very high level of detail and spatial resolution of the RGB orthophotomosaic, whereas PPAN_B_ inherits the lower level of detail and spatial resolution of the MS orthophotomosaic (Table 2).

In the case of the MS sensor MicaSense RedEdge-MX (MicaSense Inc., Seattle, WA, USA), Equation (1) is used unchanged. For the MS sensor Sequoia+ (Parrot S.A., Paris, France), where no Blue band is available, the Blue term in Equation (1) is set to zero and, by assumption, the Green and Red weights are set to 0.644 (=0.587 + 0.114/2) and 0.356 (=0.299 + 0.114/2), respectively. This weight redistribution is adopted as a reproducible heuristic to account for the absence of the Blue band, while keeping the weights summing to 1 so that the intensity scale remains unchanged.

Because the Sequoia+ (Parrot S.A., Paris, France) sensor does not record a Blue band, the resulting visible-band intensity cannot be interpreted as a physically complete luminance representation. Consequently, some sensitivity of spectral similarity metrics, including correlation and spectral angle, to this redistribution assumption is expected.

The redistribution adopted here does not aim to reconstruct physical luminance, but to produce a reproducible operational intensity proxy within the PCA substitution framework while preserving unit-sum scaling. A formal spectral response function (SRF)-based coefficient derivation would involve convolution of the sensor-specific spectral response functions with the multispectral bandpasses in order to derive physically grounded weighting coefficients. While such modelling is feasible in principle, it would shift the methodological scope of the present study toward full cross-sensor radiometric harmonization. The present study instead targets operational spectral compatibility within a substitution framework, focusing on relative spectral consistency rather than absolute radiometric equivalence. Consequently, explicit SRF-based coefficient derivation is not a prerequisite for the validity of the proposed intensity reconstruction, which operates within a statistically normalized intensity domain. The potential impact of the redistribution assumption is partially moderated by the subsequent mean–std normalization and hybrid integration with RGB-derived spatial detail, which constrain the intensity contribution within a standardized statistical space. SRF-driven coefficient derivation and dedicated sensitivity analysis are therefore framed as methodological extensions for future research rather than prerequisites for the validity of the proposed approach.

### 2.4. Resampling of PPAN_B_ and Generation of the PPAN_C_ Orthophotomosaic

To bring the PPAN_B_ orthophotomosaic onto the grid of the PPAN_A_ orthophotomosaic, PPAN_B_ was resampled using cubic interpolation [43]. This is an important step, because, unlike nearest neighbour or bilinear interpolation, cubic interpolation does not replicate the same grey level across all newly created (sub-)pixels in the resulting PPAN_C_ orthophotomosaic (Figure 2 and Figure 3) that originate from a single low-spatial-resolution PPAN_B_ pixel. Instead, it computes intermediate values that follow a smooth transition curve between neighbouring pixels, producing a continuous and visually realistic gradation of grey tones. In this way, the (sub-)pixels of the PPAN_C_ orthophotomosaic, now on a finer grid (smaller pixel size), acquire varying brightness values, thereby avoiding blockiness that would otherwise lead to pronounced pixelation in the final fused image. Within the resampling options available in the processing environment (nearest neighbour, bilinear and cubic convolution), cubic interpolation was selected as the most suitable compromise for the present application. Nearest neighbour interpolation preserves original pixel values but produces blocky transitions and pronounced pixelation when upsampling multispectral-derived intensity layers. Bilinear interpolation yields smoother transitions but introduces noticeable blurring of fine spatial gradients. Cubic convolution provides a balanced trade-off, maintaining spatial continuity while limiting both blockiness and excessive smoothing, and was therefore retained for all resampling operations. Resampling with cubic interpolation improves the visual appearance, but it does not introduce new, genuine spatial information or detail beyond what is already contained in the original MS-derived PPAN_B_.

### 2.5. Linear Combination and Normalization for Generating the PPAN_D_ Orthophotomosaic

To enable the controlled incorporation of the high-spatial-resolution detail provided by the PPAN_A_ orthophotomosaic, together with the spectrally compatible intensity of the PPAN_C_ orthophotomosaic in the visible part of the MS orthophotomosaic, an equal-weight linear combination of the two images is applied (Equation (2)).

(2) $${PPAN}_{D}=0.5\cdot (\frac{{PPAN}_{A}-\mu_{A}}{\sigma_{A}})+0.5\cdot (\frac{{PPAN}_{C}-\mu_{C}}{\sigma_{C}})$$

where μ and σ denote the mean value and standard deviation of the pixel intensities of each respective image, computed over the full spatial extent of each orthophotomosaic using valid pixels only (excluding NoData or undefined values). Prior to normalization, PPAN_A_ and PPAN_C_ are restricted to their common spatial overlap to ensure consistent statistical scaling.

Equation (2) was also examined using non-equal coefficient values, with a and b varying from 0.2 to 0.8, their sum constrained to unity, and with the consideration that values of b greater than a favour improved spectral compatibility of PPAN_D_ (Figure 2 and Figure 3) with the MS orthophotomosaic. The convergence of the spatial and spectral behaviour of the PPAN_D_ orthophotomosaic was evaluated through quantitative indicators and visual inspection. The experimental results showed that equal participation (a = b = 0.5) provides stable and balanced integration of spatial and spectral information.

Across the tested coefficient range, lower a values (i.e., stronger PPAN_C_ contribution) increased spectral similarity but reduced spatial sharpness, whereas higher a values favoured spatial detail at the expense of spectral compatibility. Equal weighting therefore emerged as the most stable compromise, ensuring consistent behaviour across study sites without biasing the fusion towards either spatial or spectral dominance.

An equal-weight linear combination does not imply equal contribution in terms of spatial or spectral content. Rather, it ensures that both images enter the result without one numerically dominating the other. In practice, the PPAN_A_ orthophotomosaic contributes primarily high spatial detail, whereas the PPAN_C_ orthophotomosaic transfers spectrally compatible intensity derived from the visible bands of the MS orthophotomosaic.

Selecting a linear combination, as opposed to non-linear or simple additive approaches, ensures that the final PPAN_D_ orthophotomosaic retains the statistical coherence, proportionality of intensities, and linearity required by PCA-based image fusion [44,45,46].

It should be noted that the sequence of intensity formulations presented in Section 2.2, Section 2.3, Section 2.4, Section 2.5 and Section 2.6 (PPAN_A_, PPAN_C_, PPAN_D_, and PPAN_E_) forms a structured reconstruction pipeline that also functions as an ablation sequence. Each formulation isolates a specific component of the reconstruction problem. PPAN_A_ represents the conventional RGB-derived intensity with strong spatial detail but limited spectral compatibility with the multispectral dataset. PPAN_C_ represents a multispectral-visible-derived intensity with maximal spectral compatibility but without genuine high-resolution spatial information. PPAN_D_ introduces statistically normalized hybrid integration of the two intensity sources. PPAN_E_ finally incorporates controlled high-frequency enhancement in order to restore spatial sharpness while maintaining the spectral behaviour imposed by the hybrid intensity. Evaluating these intermediate formulations therefore allows the necessity of each reconstruction stage to be assessed within the same experimental framework.

Before applying the equal-weight linear combination of PPAN_A_ and PPAN_C_ orthophotomosaics, statistical normalization (mean–std normalization) is performed to match the dynamic range and align the mean (μ) and standard deviation (σ) of the two orthophotomosaics (Table 3). Because these orthophotomosaics originate from different sensors and exhibit markedly different brightness and contrast distributions, directly combining them would lead to numerical imbalance, with one image dominating due to a wider value range or higher variance. Through normalization, both images are mapped into a common statistical framework, enabling stable integration of spatial and spectral information in the PPAN_D_ orthophotomosaic without distortion.

### 2.6. Spatial Enhancement Using High-Pass Filtering and Unsharp Masking for Generating the PPAN_E_ Orthophotomosaic

Given that the PPAN_D_ orthophotomosaic already has good spatial resolution, a non-aggressive enhancement of its fine detail is adopted, which leads to the use of a 3 × 3 high-pass kernel (Equation (3)).

(3) $${PPAN}_{D,HP}={PPAN}_{D}*\left(High\;pass\;kernel\;3\times 3\right)$$

when $High\;pass\;kernel\;3\times 3=\left[\begin{array}{ccc} -1 & -1 & -1\\ -1 & 8 & -1\\ -1 & -1 & -1 \end{array}\right]$ and ∗ denotes 2D convolution.

This filter and operator size suppress the low spatial frequencies of the PPAN_D_ orthophotomosaic and enhance high-frequency content (edges and fine textures) at a scale of approximately 1–2 pixels. In essence, the same underlying principle is followed as in multi-resolution analysis (MRA)-based pansharpening techniques, such as the Laplacian Pyramid, where high-frequency information is extracted from a high-spatial-resolution image and then injected in a controlled manner into a lower-spatial-resolution image [47,48]. In the present paper, high-frequency information refers to spatial sharpness and edge/texture detail and does not imply an increase in true physical spatial resolution beyond that provided by the RGB product. Accordingly, detail is enhanced in the PPAN_D_ orthophotomosaic through mild, controlled sharpening of edges and textures. The effect of this enhancement on spectral consistency is not assumed a priori but is subsequently evaluated quantitatively using correlation and SAM on the fused products. Larger operators (e.g., 5 × 5) act over broader neighbourhoods and may induce overshoot and ringing around edges, as well as alter overall brightness and object appearance.

However, the extracted high-frequency content contained in the PPAN_D,HP_ orthophotomosaic (Figure 2 and Figure 3) should not be used directly; instead, it should be incorporated into the PPAN_D_ orthophotomosaic in a controlled way using the unsharp masking technique [49,50] (Equation (4)).

(4) $${PPAN}_{E}={PPAN}_{D}+\left(\lambda \;\cdot \;{PPAN}_{D,HP}\right)$$

where *λ* is the sharpening gain. This allows only the high-frequency content, i.e., edges and fine detail, to be added, without altering the PPAN_D_ orthophotomosaic, which governs the overall brightness and the spectral behaviour of the orthophotomosaic.

Choosing *λ* values between 0.05 and 0.2 ensures non-aggressive spatial enhancement, preventing edge over-sharpening and undue changes in the statistical distribution of intensities (for λ < 0.05 the sharpening is barely noticeable, whereas for λ > 0.2 excessive edge enhancement was observed, resulting in bright outlines around objects and/or increased image noise). In this way, unsharp masking acts as a controlled mechanism for increasing spatial sharpness, with limited impact on overall brightness and on the spectral consistency of the intensity layer, as reflected by correlation and SAM in the final fused product. Consequently, the resulting PPAN_E_ orthophotomosaic (Figure 2 and Figure 3) exhibits improved spatial clarity while remaining spectrally consistent and suitable for use in the subsequent image fusion stage. For all study sites, *λ* = 0.2 was used to ensure consistent cross-site comparisons and to avoid reliance on site-specific parameter tuning. This value corresponds to the upper bound of the empirically tested gain interval, representing the highest sharpening intensity that avoids pronounced haloing or overshoot artefacts while maintaining stable intensity statistics. A limited post hoc sensitivity check was conducted using the same evaluation protocol applied throughout the study (SAM on the MS grid; spatial sharpness on the native output). The analysis indicated that increasing λ improves spatial sharpness but progressively increases spectral angle deviation beyond *λ* = 0.2, reflecting a growing spatial–spectral trade-off. Larger kernels (5 × 5) further increased high-frequency energy but introduced wider edge responses and more pronounced halo artefacts. The 3 × 3/*λ* = 0.2 configuration was therefore retained as the most stable spatial–spectral compromise under the adopted evaluation framework. All processing steps rely on explicitly defined linear transformations and fixed parameter settings, enabling straightforward reproducibility across different datasets and acquisition conditions.

### 2.7. Image Fusion with PPAN_E_ and MS Orthophotomosaic

PCA was selected as the fusion method, as it is a standard component substitution (CS) technique [51,52,53]. PCA transforms the MS channels into principal components, where the first component (PC1) captures the largest share of the total variance and mainly reflects overall brightness differences in the image. PC1 is then replaced by PPAN_E_, followed by an inverse transform to produce a new multispectral product at the spatial resolution of PPAN_E_. No histogram matching or moment-based radiometric normalization was applied prior to substitution. The PPAN_E_ component is introduced directly in order to preserve the explicit radiometric contribution of the enhanced intensity signal within the PCA space. Any radiometric or spectral effects arising from this substitution are therefore evaluated empirically through downstream spectral similarity metrics, including correlation and spectral angle mapping. In addition, classical component substitution fusion methods (Multiplicative and Brovey transforms) were applied to the same co-registered datasets to enable direct comparative evaluation.

To support the assumption that the first principal component (PC1) approximates a common intensity/brightness-like component of the MS image, the output files (Site_eigenvalues.tbl and Site_eigenmatrix.mtx; one file pair for each of the seven archaeological sites) from Erdas Imagine Professional 2023© v.16.8.1 (Hexagon AB, Stockholm, Sweden) were used. Specifically, the proportion of total MS variance captured by PC1 was computed (EV_PC1 = eigenvalue_1/Σeigenvalues), along with the PC1 loadings for each MS band (first column of the eigenmatrix). As shown in Table 4, PC1 accounts for the largest proportion of total variance across all sites (68.92–90.60%), while the PC1 loadings share the same sign and are comparable in magnitude, indicating that PC1 primarily represents the common intensity component of the MS dataset. Therefore, substituting PC1 with an intensity image is consistent with the standard rationale of PCA-based component substitution, since PC1 acts as the main carrier of the shared intensity information in the MS dataset.

### 2.8. Quantitative Spectral and Spatial Assessment

A common protocol was adopted to evaluate the fused products, covering both spectral consistency and spatial sharpness. Spectral assessment was based on band-wise correlations and the Spectral Angle Mapper (SAM), while spatial sharpness was evaluated on the NIR band using the Tenengrad sharpness index and Laplacian variance.

For strict pixel-to-pixel comparison in the spectral assessment, all correlation and SAM computations were carried out on the MS grid. Specifically, the fused products from all fusion variants (PPAN_A_, PPAN_C_, PPAN_E_) were resampled onto the MS grid so that comparisons were performed at the same pixel size, spatial extent, and alignment. Metrics were computed only for pixels with valid values (i.e., excluding NoData/NaN) in both datasets (MS and fused image). Spatial sharpness was computed on the original (native) grid of the fused products (RGB resolution), using only pixels with valid values across all three fused products.

It is noted that a classical full-reference reduced-resolution protocol, or indices such as ERGAS (Erreur Relative Globale Adimensionnelle de Synthèse) and QNR (Quality with No Reference), are not employed, because in the present applications there is no true PAN band and the intensity layer is reconstructed (PPAN) from a different sensor with different spectral responses. The multi-sensor incompatibility and the required statistical adjustments (mean–std normalization and sharpening) violate the assumptions underlying standard protocols.

#### 2.8.1. Spectral Assessment—Correlation and SAM

Fusion quality was evaluated using correlation matrices between the original MS bands and the corresponding bands of the fused products across the seven archaeological sites (Table 5). To provide a compact quantitative summary of Table 5, the mean corresponding-band correlation across all sites increases from 0.842 (PPAN_A_) to 0.928 (PPAN_E_). Averaged per band, the largest gains are observed in the Red Edge band (+0.106), followed by Green (+0.091). At the site level, the strongest improvements occur at Kasta Tumulus and Olynthus. No systematic degradation of spectral similarity metrics was observed between RedEdge-MX and Sequoia+ subsets, indicating that the proposed intensity reconstruction remains robust despite cross-sensor spectral response differences. To assess whether the observed across-site spectral improvements are statistically robust, paired non-parametric Wilcoxon signed-rank tests were conducted across the seven sites using site-mean values. The increase in mean corresponding-band correlation from PPAN_A_ to PPAN_E_ was statistically significant (two-sided Wilcoxon signed-rank test, *p* = 0.0156), with a large effect (r = 0.91) and maximum rank-biserial correlation (RBC = 1.00), indicating consistent improvement across all sites. For SAM, the two-sided paired test did not reach the 0.05 significance level (*p* = 0.0781), reflecting limited power at N = 7; under the directional hypothesis of reduced spectral-angle deviation, a one-sided paired test supports a significant reduction (*p* = 0.0391), with a large effect (r = 0.67) and strong rank-biserial correlation (RBC = 0.79). In general, correlation values > 0.9 indicate high spectral similarity between the corresponding bands of the original MS image and the fused image. The correlation analysis focuses on corresponding-band relationships in order to evaluate spectral preservation fidelity. Off-diagonal correlations, which may reflect inter-band mixing effects, were not analysed explicitly, as the present assessment framework prioritises band-preservation performance rather than cross-band leakage characterization. In this paper, high correlations are interpreted as evidence of preservation of the relative spectral behaviour of the bands, rather than as a comprehensive assessment of all possible radiometric errors.

Beyond the fusions based on the original PPAN_A_ and the new PPAN_E_, an additional control experiment was also carried out, in which PPAN_C_ alone was used as the intensity image in the PCA, i.e., a product derived from the visible bands of the MS orthophotomosaic and resampled onto the grid of the RGB orthophotomosaic. This fusion variant (using PPAN_C_) was executed with the same PCA procedure (same MS bands and the same PC1 substitution) so as to function as a control. PPAN_C_ is an MS-visible-derived intensity and, although resampled to the RGB grid, it does not introduce genuine new spatial detail (since the increase in resolution results from interpolation). In this way, comparing PPAN_C_ with PPAN_E_ supports the role of the PPAN_E_ orthophotomosaic as a balanced solution between spectral similarity and spatial sharpness.

For strict pixel-by-pixel comparison in the spectral assessment, all correlation and SAM computations were performed on the MS grid. Specifically, the fused products (RGB resolution) were resampled to the MS grid using the same interpolation method for all fusion variants (using PPAN_A_, PPAN_C_, and PPAN_E_), so that comparisons were made at the same pixel size/extent/alignment. Metrics were computed only where both the MS data and the resampled fused product have valid values (i.e., excluding NoData/NaN). Spatial sharpness was assessed on the original grid of the fused products, i.e., at RGB resolution, using only pixels with valid values in all three fused products.

In addition to the correlation matrices, the Spectral Angle Mapper (SAM) index [19,20,21], a standard measure of spectral distortion, was computed. SAM measures the angular distance between the spectral vector of a pixel in the original MS orthophotomosaic and the corresponding pixel in the fused product (Equation (5) [19,20,21]).

(5) $$SAM\left(x\right)=arccos(\frac{M\left(x\right)\cdot \;F\left(x\right)}{\left\|M\left.\left(x\right)\right\|\right.\;\cdot \;\left\|F\left.\left(x\right)\right\|\right.})$$

with the Euclidean norm $\left\|M\left.\left(x\right)\right\|\right.=\sqrt{\sum_{i=1}^{Β}M_{i}{\left(x\right)}^{2}}$ and $\left\|F\left.\left(x\right)\right\|\right.=\sqrt{\sum_{i=1}^{Β}F_{i}{\left(x\right)}^{2}}$ where $M\left(x\right)={\left[M_{1}\left(x\right),\;\ldots ,\;M_{Β}\left(x\right)\right]}^{Τ}$ is the spectral vector of the MS bands at pixel x, $F\left(x\right)={\left[F_{1}\left(x\right),\;\ldots ,\;F_{Β}\left(x\right)\right]}^{Τ}$ is the corresponding vector of the fused product at the same pixel x, and Β is the number of bands.

Overall, SAM results are reported in Table 6, with a concise across-site summary in Table 7. For each site and each fusion variant (using PPAN_A_, PPAN_C_, and PPAN_E_), a per-pixel SAM raster was computed on the MS grid, considering only pixels with valid values (without NoData/NaN). Summary statistics were then extracted from the SAM distribution per site, i.e., Mean and Median, as well as the threshold below which 95% of SAM values (valid pixels) fall for each site and are presented in Table 6. The median is additionally reported as a robust summary measure to reduce the influence of outliers. Table 7 is derived directly from Table 6; i.e., for each fusion variant, the site-level mean SAM is taken as the Mean value in Table 6 for each of the seven sites, and the across-site Mean, standard deviation (Std), and Median are then computed (unweighted, one value per site), thereby assigning equal weight to each study site irrespective of spatial extent or pixel count. Across-site averages are therefore reported as unweighted means, treating each site as an independent experimental unit. Given that the objective of the study is to evaluate methodological robustness across heterogeneous acquisition contexts rather than to derive landscape-scale radiometric generalizations, equal weighting is considered appropriate. Pixel-weighted aggregation would primarily reflect scene extent rather than fusion behaviour and is therefore not adopted as the primary comparative indicator. An exploratory pixel-weighted aggregation was additionally computed across all valid pixels per site. The resulting differences relative to the equal-site averaging strategy were minimal (<0.02 in correlation-equivalent terms and <0.3° in SAM) and did not alter the relative ranking of fusion variants. At the site level (site-level mean SAM), PPAN_E_ shows a lower mean SAM than PPAN_A_ in five of the seven sites, Sanctuary of Eukleia, Kasta Tumulus, Acropolis of Platania, Early Christian Basilica C, and Olynthus, indicating reduced spectral-angle distortion in most cases.

Two exceptions are observed (Ancient Theatre of Mieza and Mosaic at Dion), where the mean SAM of PPAN_E_ is slightly higher than that of PPAN_A_ (Table 6). This deviation is consistent with the fact that PPAN_E_ incorporates high spatial frequencies in a controlled manner through intensity sharpening, which in certain scenes/textures can increase the per-pixel angular distance of spectral vectors even when band-wise correlations remain high. The two sites exhibiting increased SAM (Ancient Theatre of Mieza and Mosaic at Dion) are characterized by high micro-texture density and strong reflectance contrast transitions. In such scenes, controlled high-frequency injection increases spectral vector angular dispersion despite preserved inter-band correlation structure. It is noted that, for the Mosaic at Dion, the difference is very small, whereas at the Ancient Theatre of Mieza the result highlights that the spatial-enhancement–spectral-distortion trade-off is not zero, although it is generally favourable for the majority of sites.

At the overall level (Table 7), PPAN_E_ yields a lower mean site-mean SAM than PPAN_A_ (4.264° versus 5.782°) and a lower median (3.984° versus 6.358°), reinforcing the conclusion that PPAN_E_ improves overall (across-sites) spectral preservation relative to PPAN_A_, beyond the correlation-based assessment. At the same time, as expected for an MS-derived intensity control, PPAN_C_ achieves the lowest overall SAM (mean 3.126°, median 2.805°).

#### 2.8.2. Spatial Sharpness—Tenengrad and Laplacian Variance in the NIR

In addition to the spectral assessment of the fused products (correlation and SAM), a quantitative evaluation of spatial sharpness was also performed, to demonstrate that the proposed PPAN_E_ orthophotomosaic not only improves spectral consistency but also preserves the high spatial sharpness of the RGB orthophotomosaic. This analysis is necessary because the examples shown in Figure 2 and Figure 3, while indicative, remain qualitative and are insufficient as quantitative evidence.

The assessment was carried out on the NIR band of the fused products, as NIR is a band of particular importance and is also sensitive to the transfer of information through fusion techniques. Spatial sharpness was evaluated at the original resolution of the fused products (i.e., at RGB resolution), so that the metrics reflect sharpness at the spatial scale at which the fused result is produced. To ensure comparability, measurements were computed only for pixels with valid values in all three fused products (using PPAN_A_, PPAN_C_, and PPAN_E_), so that all metrics were derived from the same pixels.

For each site, three square AOIs of approximately 500 × 500 pixels (3–7 m per side, depending on the RGB ground sampling distance reported in Table 2) were selected in edge-rich areas containing strong structural boundaries and fine textures (e.g., masonry edges, material transitions). Homogeneous, shadowed, and saturated regions were avoided. Identical AOI footprints were applied across all fusion variants (PPAN_A_, PPAN_C_, and PPAN_E_) to ensure direct comparability. All sharpness metrics were computed on the fused NIR band on the native RGB grid using only pixels with valid values across all products. Within each AOI normalization was applied (Equation (6) [24,25]). Because spatial sharpness is evaluated on three AOIs per site, the sampling design remains spatially selective; therefore, the spatial comparison is interpreted as indicative of consistent sharpness trends rather than as a basis for formal statistical generalization across entire sites. To further evaluate potential sampling bias, additional randomly positioned AOIs were tested on two representative sites. The relative ranking of fusion variants in terms of Tenengrad and Laplacian variance remained unchanged, confirming that the edge-focused AOI selection did not alter the comparative conclusions regarding spatial sharpness performance.

(6) $$I_{z}\left(x\right)=\frac{I\left(x\right)-\mu_{I}}{\sigma_{I}}$$

where I(x) is the fused NIR value at pixel x and $\mu_{I}$, $\sigma_{I}$ are the mean and standard deviation of values within the specific AOI. This normalization helps isolate spatial sharpness from differences in contrast or dynamic range.

Two spatial sharpness metrics were then computed. The first (Table 8) is Tenengrad, defined as the mean gradient magnitude of the image computed using Sobel operators (Equation (7) [22,23]).

(7) $$T=\frac{1}{N}\sum_{x\in AOI}\sqrt{(G_{x}(x){)}^{2}+(G_{y}(x){)}^{2}}$$

where $G_{x}$ and $G_{y}$ are the horizontal and vertical Sobel derivatives, and N is the number of pixels in the AOI. This metric captures edge energy and increases as spatial sharpness is enhanced.

The second metric (Table 8) is the variance $V_{L}$ of the Laplacian response (Equation (8) [23,26,27,28]).

(8) $$V_{L}={Var}_{x\in AOI}\left(I_{z}\left(x\right)*K_{\nabla^{2}}\right)={Var}_{x\in AOI}\left(R\left(x\right)\right)=\frac{1}{N}\sum_{x\in AOI}({R\left(x\right)-\bar{R})}^{2}$$

where $K_{\nabla^{2}}$ is a 3 × 3 Laplacian kernel, ∗ denotes 2D convolution, and N is the number of pixels in the AOI. The variance $V_{L}$ captures high-spatial-frequency energy and is widely used as an indicator of detail/sharpness.

To verify that the observed spatial behaviour is not restricted to the NIR band, supplementary sharpness measurements were conducted on the Red band of the fused products using the same AOI-based protocol (z-score normalization within AOIs; Tenengrad and Laplacian variance computed on the native fused grid). The relative ranking among fusion variants remained unchanged across tested sites and sensors. For example, on representative RedEdge-MX and Sequoia+ datasets, PPAN_E_ retained sharpness values close to PPAN_A_ and substantially higher than PPAN_C_ (e.g., Tenengrad 2.30 vs. 0.47 and Laplacian variance 6.91 vs. 0.37 on a RedEdge site; Tenengrad 1.74 vs. 0.43 and Laplacian variance 3.91 vs. 0.03 on a Sequoia site). This confirms that the spatial–spectral balance observed in the NIR generalizes to the visible range under the adopted fusion framework.

To further ensure that the observed spatial behaviour does not introduce sharpening artefacts within the visible range, halo/overshoot behaviour was quantified on the Red band, selected as a representative high-contrast visible channel. Edge pixels were detected using a Sobel-gradient mask, and overshoot was defined when the centre pixel exceeded the local 8-neighbour extrema by more than 5% of the robust local dynamic range. Across representative RedEdge-MX and Sequoia+ subsets, PPAN_E_ exhibited low overshoot ratios (0.33–1.72%), remaining well below this threshold, while PPAN_A_ remained near-zero (0.03–0.15%). These findings indicate that the adopted 3 × 3/*λ* = 0.2 sharpening enhances spatial detail without inducing pronounced halo artefacts.

To further position the proposed pseudo-panchromatic reconstruction within the broader pansharpening framework, additional fusion experiments were conducted using established component substitution techniques. Specifically, Multiplicative and Brovey transforms were applied to the same co-registered RGB and multispectral orthophotomosaic, following the identical preprocessing and evaluation protocol described above.

For consistency, spectral metrics (band-wise correlation and SAM) were computed on the MS grid, while spatial metrics (Tenengrad and Laplacian variance) were derived from the NIR band of the fused products at native resolution.

To provide a consolidated comparative assessment, performance metrics were aggregated across the seven archaeological sites. The cross-site mean and standard deviation values for each fusion method are summarized in Table 9.

The aggregated results indicate that purely MS-derived intensity substitution (PPAN_C_) achieves the highest spectral similarity but exhibits severe spatial degradation. The conventional RGB-derived PPAN_A_ approach preserves spatial sharpness but demonstrates lower spectral consistency.

**Figure 4:**
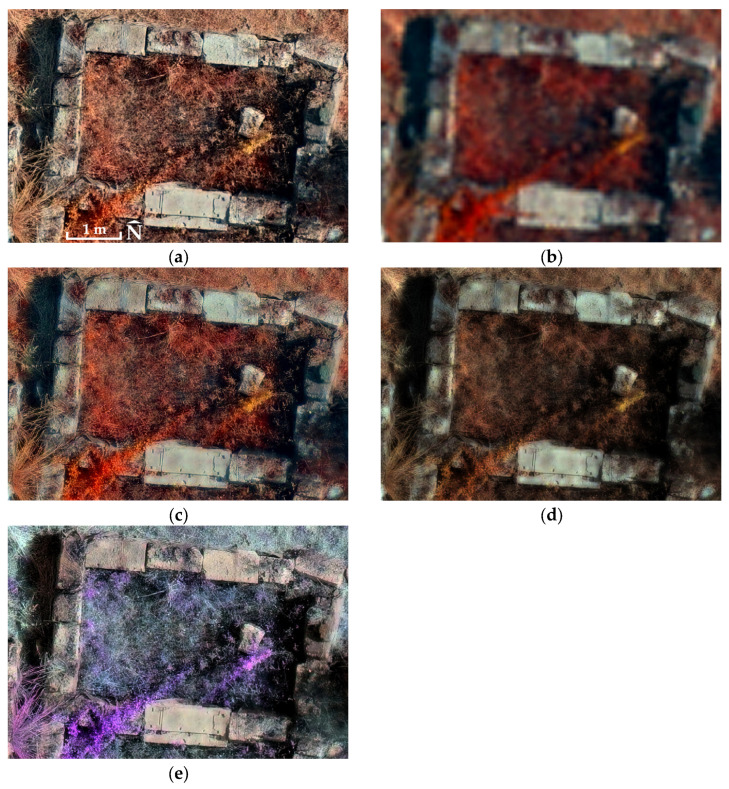
Comparative fusion outputs for the Sanctuary of Eukleia: (**a**) PCA–PPAN_A_; (**b**) PCA–PPAN_C_; (**c**) PCA–PPAN_E_; (**d**) Multiplicative; (**e**) Brovey. Native-resolution excerpts illustrating spatial and spectral differences among fusion approaches. Centre of images 40°28′47.34″ N 22°19′17.76″ E.

**Figure 5:**
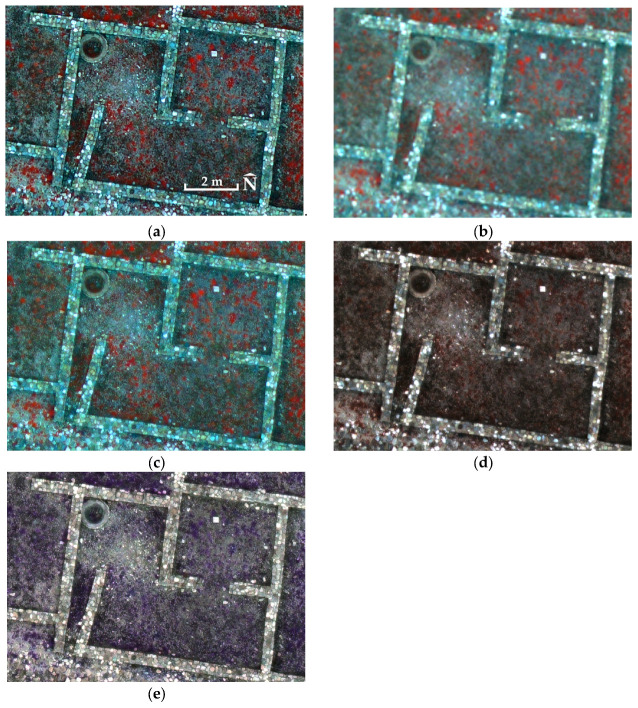
Comparative fusi. outputs for Olynthus: (**a**) PCA–PPAN_A_; (**b**) PCA–PPAN_C_; (**c**) PCA–PPAN_E_; (**d**) Multiplicative; (**e**) Brovey. Native-resolution excerpts illustrating spatial and spectral differences among fusion approaches. Centre of images 40°17′46.13″ N 23°21′16.97″ E.

The proposed PPAN_E_ intensity reconstruction provides a balanced performance profile, improving spectral behaviour relative to PPAN_A_ while maintaining spatial sharpness close to RGB-driven substitution. Classical component substitution methods such as Multiplicative and Brovey fusion show competitive spectral metrics but do not achieve the same spatial–spectral balance under cross-sensor RGB–MS conditions, and these quantitative differences are also visually supported by the comparative excerpts presented in Figure 4 and Figure 5.

The visual differences observed in Figure 4 and Figure 5 are consistent with the quantitative findings reported in Table 9. While MS-derived intensity substitution enhances spectral similarity, it produces visibly reduced edge clarity. In contrast, the proposed PPAN_E_ reconstruction preserves high-frequency spatial detail while mitigating spectral distortion relative to conventional RGB-derived substitution.

## 3. Discussion

Correlation provides strong evidence for preservation of relative spectral behaviour (i.e., the extent to which value fluctuations co-vary), but it does not fully capture absolute radiometric fidelity. For this reason, SAM is used as a complementary, standard measure of spectral distortion, so that the paper’s conclusion regarding improved spectral consistency is supported both by correlation-based similarity and by reduced angular deviation of spectral vectors.

While high baseline correlations (>0.9) indicate strong preservation of inter-band co-variation, correlation alone does not guarantee absence of subtle spectral distortions. The complementary use of the Spectral Angle Mapper (SAM) ensures sensitivity to angular deviations in spectral vector space, thereby jointly constraining both co-variation fidelity and spectral-shape distortion. Within the present cross-sensor UAS framework—where no true panchromatic reference exists and the substituted intensity is reconstructed rather than physically measured—classical reduced-resolution or PAN-dependent radiometric indices would require additional modelling assumptions beyond the scope of this study. The adopted evaluation strategy therefore prioritizes complementary metrics that remain methodologically consistent under multi-sensor intensity reconstruction conditions.

The paired statistical analysis across the seven sites confirms that the correlation improvement achieved by PPAN_E_ over PPAN_A_ is statistically significant. In contrast, the reduction in SAM, although observed in the majority of sites, does not reach the conventional 0.05 significance level under a two-sided paired test, reflecting the limited statistical power associated with the small number of sites (N = 7). Under a directional testing framework, consistent with the expected reduction in spectral-angle deviation, the SAM improvement is supported at the one-sided significance level. This behaviour is consistent with the known sensitivity of SAM to controlled high-frequency injection and highlights the spatial–spectral trade-off inherent in intensity-enhanced fusion.

Although RGB orthophotomosaics are DN-scaled and MS datasets are reflectance-scaled, the PPAN reconstruction operates within a standardized intensity space following mean–std normalization. This statistical normalization removes scale dependency prior to PCA substitution. Consequently, fusion behaviour is governed by relative intensity gradients rather than absolute radiometric magnitude.

Absolute radiometric harmonization of RGB reflectance was not pursued, as the evaluation framework targets relative spectral consistency rather than reflectance retrieval accuracy.

The contribution of this study does not lie in the individual processing operators employed (linear normalization, PCA substitution, or spatial sharpening), which are well established, but in their formulation as a spectrally compatible intensity reconstruction strategy designed to mitigate cross-sensor substitution distortion.

While the individual operators are standard, their integration follows a compatibility-constrained design. In particular, the reconstruction explicitly separates spectral compatibility (provided by the MS-visible-derived intensity) from spatial gradient transfer (provided by the RGB-derived intensity), and combines them within a normalized intensity space prior to controlled high-frequency injection.

This structured formulation differs from simple weighted blending approaches, where intensity layers are combined without an explicit normalization framework or a controlled separation between spectral and spatial components. The intermediate reconstructions (PPANA, PPANC, and PPAND) demonstrate that neither purely spatial intensity substitution nor purely multispectral-derived intensity is sufficient to achieve the desired spatial–spectral balance.

Spectral compatibility of the fused images was assessed by analysing correlation coefficients between the MS bands and the corresponding bands of the fused images produced using the PPAN_A_, PPAN_C_, and PPAN_E_ orthophotomosaics. Comparisons across multiple sites, spanning different environments and numbers of available bands, allow both the effectiveness and the stability of the proposed methodology to be evaluated.

The fusion using the PPAN_C_ orthophotomosaic serves as a control, because PPAN_C_ is derived from the visible bands of the MS orthophotomosaic and, although resampled to the RGB orthophotomosaic pixel size, it does not introduce new spatial resolution comparable to the RGB orthophotomosaic. Thus, the PPAN_C_ control bounds the trade-off, i.e., near-maximal spectral similarity when the intensity is MS-derived, versus genuine injection of high spatial frequencies when the intensity incorporates information from the RGB data, as in PPAN_E_ and PPAN_A_. The examples in Figure 2 and Figure 3 visually confirm that PPAN_E_ retains clearly higher spatial sharpness than PPAN_C_, while the correlation matrices (Table 5) show that PPAN_E_ consistently achieves high spectral similarity in terms of the relative, per-band variation in values. Within this comparative framework, PPAN_C_ may be interpreted as an upper bound on achievable spectral similarity under minimal spatial enhancement, whereas PPAN_E_ represents the balanced operating point that preserves most of the RGB-driven spatial detail while reducing spectral distortion relative to purely spatial fusion.

Across all sites, band-wise correlations are maintained or improved when PPAN_A_ is replaced by PPAN_E_ (Table 5). At the same time, SAM provides across-site evidence of reduced spectral-angle distortion for PPAN_E_ relative to PPAN_A_ (Table 7), while also indicating small local trade-offs at specific sites consistent with controlled spatial enhancement.

An additional noteworthy observation is that the improvement in spectral correlation is not uniform across all bands but is consistently more pronounced in the Red Edge and NIR bands than in the visible bands (Blue, Green, and Red). This is consistent with the fact that Red Edge and NIR bands are often more sensitive to spectral leakage and contrast transfer in PCA-based fusion when the intensity is not spectrally compatible with the MS orthophotomosaic.

The quantitative spatial sharpness assessment (Table 8) supports the spatial component of the proposed approach. Across all sites, the fusion variant using PPAN_C_ orthophotomosaic yields the lowest Tenengrad and Laplacian-variance values, as expected, since it does not introduce genuine new spatial information beyond interpolation. In contrast, the fusion variant using PPAN_E_ consistently produces substantially higher values than PPAN_C_, and values comparable to PPAN_A_ orthophotomosaic, indicating that the proposed PPAN_E_ preserves high spatial sharpness consistent with the RGB product. Taken together with the spectral assessment results (correlation and SAM), these findings show that PPAN_E_ achieves a meaningful balance between spatial sharpness and spectral consistency, improving the overall quality of the fused products relative to the conventional PPAN_A_ approach, a behaviour also visually supported by the comparative excerpts shown in Figure 4 and Figure 5. The comparative evaluation summarized in Table 9 further supports this interpretation, demonstrating that the proposed hybrid pseudo-panchromatic reconstruction achieves a stable spatial–spectral balance relative to established component substitution baselines. The goal of the comparative evaluation is therefore not to demonstrate universal superiority over all pansharpening approaches, but to examine the spectral–spatial behaviour of different intensity constructions within a consistent substitution framework.

## 4. Conclusions

This paper addresses a practical gap that arises systematically in UAS image-fusion applications. In most cases there is no true PAN band, while the available RGB and MS products originate from different sensors with different spectral responses and spatial resolutions. The solution of generating a PPAN_A_ from the RGB orthophotomosaic and applying PCA-based fusion is effective in terms of spatial sharpness, but it is constrained by RGB–MS spectral incompatibility, which manifests as spectral distortion and reduced spectral similarity between the fused product and the original MS data.

The evaluation across seven archaeological sites showed that using PPAN_E_ consistently improves the spectral consistency of the fused products relative to the conventional PPAN_A_ approach. Band-wise correlations between the MS and fused products increase across sites and across bands, with many values remaining stably high, while the improvement is more pronounced and more consistent in the Red Edge and NIR bands. In parallel, the SAM index indicates reduced spectral distortion for PPAN_E_ compared with PPAN_A_, both at the individual-site level and on average across all sites (Table 7). These results support the conclusion that PPAN_E_ yields fused products that better preserve the relative (correlation-based) spectral behaviour of the MS image, with reduced deviation/distortion relative to PPAN_A_. Here, spectral preservation refers to improved relative spectral consistency, as quantified through correlation and spectral-angle metrics, rather than to guaranteed absolute radiometric fidelity or reflectance reconstruction accuracy.

The study demonstrates that, in cross-sensor UAS fusion where no native panchromatic band exists, spectral distortion is governed primarily by intensity incompatibility rather than by the substitution mechanism itself. The proposed PPAN_E_ reconstruction provides a reproducible compatibility layer that bridges RGB spatial detail with multispectral spectral structure prior to fusion. The sequential evaluation of PPAN_A_, PPAN_C_, PPAN_D_, and PPAN_E_ further shows that the proposed formulation operates as a structured intensity reconstruction framework rather than as a simple weighted combination of existing intensity layers.

Alongside the spectral results, spatial sharpness was quantified on the NIR band using sharpness metrics (Tenengrad and Laplacian variance) in three selected areas per site with pronounced edges and textures, considering only the common pixels with valid values (i.e., excluding NoData/NaN). As shown in Table 8, fusion using PPAN_E_ consistently exhibits markedly higher spatial sharpness than fusion using the PPAN_C_ orthophotomosaic, and values comparable to fusion using PPAN_A_. This demonstrates that PPAN_E_ preserves spatial detail consistent with the RGB product, while at the same time (based on correlation and SAM) improving spectral consistency relative to PPAN_A_.

Across seven archaeological sites, the proposed PPANE reconstruction increased mean corresponding-band correlation by +0.086 (0.842 → 0.928) and reduced mean spectral angle by −1.52° (5.782° → 4.264°), while preserving approximately 86% of the spatial sharpness relative to RGB-derived substitution. These results indicate that the proposed intensity reconstruction provides a stable spatial–spectral compromise under cross-sensor UAS fusion conditions where no native panchromatic band is available.

The method provides a practical and reproducible intensity substitute for operational UAS PCA fusion where no native panchromatic band is available.

As future tasks, sensitivity analyses are suggested for different surface types (e.g., vegetation versus built materials), comparisons with alternative pansharpening techniques under a common evaluation framework, and extension of the assessment towards absolute radiometric fidelity using additional metrics and reduced-resolution/full-reference protocols where methodologically feasible.

## Figures and Tables

**Table 1:** Summary of flight and georeferencing information (RGB and MS), as well as the spatial resolution of the derived products (DSM and orthophotomosaic) for the seven archaeological study sites. The column indicating “without GCPs” (i.e., PPK workflow) and “with GCPs” specifies the georeferencing approach applied, while resolution values are reported as GSD (cm). For the Dion archaeological site mosaic, single-image processing was performed. Flight height denotes the nominal acquisition altitude above ground level (AGL) for each RGB and multispectral mission.

Position	Flight Heigth		Georeferencing Approach	Spatial Resolution	
	RGB	MS		DSM	Orthophotomosaic
Sanctuary of Eukleia	60 m	100 m	without GCPs	RGB 1.5 MS 13.5	RGB 0.8 MS 7.0
Ancient Theatre of Mieza	60 m	100 m	without GCPs	RGB 2.2 MS 13.5	RGB 1.0 MS 7.0
Kasta Tumulus	60 m	100 m	without GCPs	RGB 1.3 MS 14.9	RGB 0.6 MS 7.0
Acropolis of Platania	67 m	100 m	without GCPs	RGB 2.1 MS 16.7	RGB 1.0 MS 8.0
Mosaic at Dion	4 m	4 m	with GCPs	-	RGB 0.1 MS 0.4
Early Christian Basilica C	30 m	30 m	with GCPs	RGB 2.5 MS 5.6	RGB 1.2 MS 2.4
Olynthus	100 m	90 m	with GCPs	RGB 2.6 MS 12.2	RGB 1.3 MS 6.1

**Table 2:** Spatial resolution (in cm) of PPAN_A_ (from RGB image) and PPAN_B_ (from MS image).

Position	PPAN_A_	PPAN_B_
Sanctuary of Eukleia	0.8	7.0
Ancient Theatre of Mieza	1.0	7.0
Kasta Tumulus	0.6	7.0
Acropolis of Platania	1.0	8.0
Mosaic at Dion	0.1	0.4
Early Christian Basilica C	1.2	2.4
Olynthus	1.3	6.1

**Table 3:** Mean (μ) and standard deviation (σ) of brightness for the PPAN_A_ and PPAN_C_ images. The different value scales reflect the fact that MS orthophotomosaics are used in per-band reflectance scale (as produced by panel-based calibration), whereas RGB orthophotomosaics are used as DN-scaled (16-bit) products.

Position	PPAN_A_		PPAN_C_	
	μ	σ	μ	σ
Sanctuary of Eukleia	30,388.266	11,837.447	65.262	29.471
Ancient Theatre of Mieza	2100.354	1005.153	64.393	32.502
Kasta Tumulus	3064.205	1765.578	36.359	23.853
Acropolis of Platania	13,356.491	7545.194	41.534	29.413
Mosaic at Dion	31,371.076	6558.637	39.148	35.113
Early Christian Basilica C	11,573.239	5957.549	52.446	35.380
Olynthus	3349.318	1336.937	52.238	29.018

**Table 4:** PCA diagnostics per site. Explained variance (EV) of PC1 and PC1 loadings per MS band (Bands Blue, Green, Red, RedEdge, NIR). For Sequoia+ datasets, the Blue band is not available: “—” indicates that no value is available.

Position	Bands	EV(PC1) %	Blue	Green	Red	RedEdge	NIR
Sanctuary of Eukleia	5	83.05	0.323	0.293	0.273	0.519	0.686
Ancient Theatre of Mieza	5	76.64	0.174	0.164	0.176	0.441	0.848
Kasta Tumulus	5	82.41	0.439	0.452	0.460	0.447	0.434
Acropolis of Platania	5	73.60	0.455	0.450	0.450	0.443	0.434
Mosaic at Dion	4	90.60	—	0.152	0.094	0.487	0.856
Early Christian Basilica C	4	68.92	—	0.558	0.566	0.520	0.310
Olynthus	5	80.97	0.355	0.351	0.351	0.433	0.654

**Table 5:** Correlation between each MS band and its corresponding fused band (diagonal values) for the seven archaeological sites, comparing PCA fusion results using three intensity images: PPAN_A_, PPAN_C_ and PPAN_E_ (Bands 1 = Blue, 2 = Green, 3 = Red, 4 = Red Edge and 5 = NIR; Blue band not available for Sequoia+ datasets).

Position			Fused Image with PPAN_A_						Fused Image with PPAN_C_						Fused Image with PPAN_E_					
		Bands	1	2	3	4	5	Mean	1	2	3	4	5	Mean	1	2	3	4	5	Mean
Sanctuary of Eukleia	MS	1	0.88						0.96						0.93					
		2		0.82						0.94						0.90				
		3			0.80						0.94						0.90			
		4				0.86						0.92						0.95		
		5					0.87						0.96						0.96	
	Mean							0.846						0.944						0.928
Ancient Theatre of Mieza	MS	1	0.91						0.97						0.96					
		2		0.84						0.94						0.93				
		3			0.91						0.97						0.96			
		4				0.77						0.88						0.90		
		5					0.83						0.83						0.95	
	Mean							0.852						0.918						0.940
Kasta Tumulus	MS	1	0.82						0.99						0.91					
		2		0.78						0.98						0.92				
		3			0.79						0.99						0.92			
		4				0.76						0.97						0.92		
		5					0.86						0.98						0.95	
	Mean							0.802						0.982						0.924
Acropolis of Platania	MS	1	0.85						0.93						0.89					
		2		0.78						0.91						0.88				
		3			0.83						0.92						0.89			
		4				0.79						0.92						0.88		
		5					0.86						0.98						0.95	
	Mean							0.822						0.932						0.898
Mosaic at Dion	MS	1	-						-						-					
		2		0.92						0.96						0.96				
		3			0.90						0.93						0.95			
		4				0.89						0.94						0.93		
		5					0.88						0.97						0.94	
	Mean							0.898						0.950						0.945
Early Christian Basilica C	MS	1	-						-						-					
		2		0.90						0.98						0.96				
		3			0.92						0.98						0.96			
		4				0.85						0.96						0.92		
		5					0.92						0.96						0.97	
	Mean							0.898						0.970						0.953
Olynthus	MS	1	0.79						0.97						0.90					
		2		0.77						0.97						0.90				
		3			0.78						0.98						0.91			
		4				0.77						0.97						0.93		
		5					0.90						0.99						0.96	
	Mean							0.802						0.976						0.920
Mean per band		1	0.850						0.964						0.918					
		2	0.830						0.954						0.921					
		3	0.847						0.959						0.927					
		4	0.813						0.937						0.919					
		5	0.874						0.953						0.954					
Overall mean			0.842						0.953						0.928					

**Table 6:** Spectral Angle Mapper (SAM, degrees) between the original MS and the fused products (PCA using PPAN_A_, PPAN_C_, PPAN_E_). Values are reported in degrees as: Mean—(Median)—[upper-tail value], where the bracketed value is defined as the threshold below which 95% of valid pixels in the site-specific SAM distribution fall. Lower values indicate smaller spectral deviation (spectral distortion). Blue band not available for Sequoia+ datasets.

Position	Bands	PPAN_A_	PPAN_C_	PPAN_E_
Sanctuary of Eukleia	5	2.823—(2.538)—[5.771]	2.155—(1.679)—[5.529]	1.311—(0.979)—[3.432]
Ancient Theatre of Mieza	5	3.304—(2.769)—[8.043]	1.958—(1.376)—[6.724]	3.984—(3.174)—[11.323]
Kasta Tumulus	5	7.140—(6.762)—[12.595]	2.805—(2.139)—[7.251]	3.676—(2.979)—[9.058]
Acropolis of Platania	5	7.104—(5.422)—[16.623]	5.070—(3.677)—[12.604]	5.278—(3.722)—[13.918]
Mosaic at Dion	4	2.018—(1.702)—[4.916]	1.896—(2.028)—[7.572]	2.107—(1.616)—[5.068]
Early Christian Basilica C	4	6.358—(5.008)—[15.761]	3.336—(1.673)—[12.635]	4.645—(3.781)—[12.075]
Olynthus	5	11.726—(10.826)—[18.677]	4.664—(3.759)—[10.042]	8.844—(7.871)—[17.669]

**Table 7:** Across-sites summary for the seven sites. Values are reported in degrees as the Mean/Std/Median of the site-level mean SAM for each fusion variant.

Method	Mean of Site-Mean SAM	Std Across Sites	Median of Site-Mean SAM
PPAN_A_	5.782	3.374	6.358
PPAN_C_	3.126	1.298	2.805
PPAN_E_	4.264	2.448	3.984

**Table 8:** Quantitative assessment of spatial sharpness in the NIR band of the fused products obtained via PCA fusion using PPAN_A_, PPAN_C_, and PPAN_E_ as the intensity input. Values are reported as mean ± standard deviation (mean ± std) of the Tenengrad (Ten) and Laplacian variance (Lap) metrics across the three AOIs per site. The Ten columns represent edge energy (gradient-based sharpness), whereas the Lap columns represent high-frequency energy (fine-detail content). Higher values indicate increased spatial sharpness.

Position	Bands	Ten PPAN_A_	Ten PPAN_C_	Ten PPAN_E_	Lap PPAN_A_	Lap PPAN_C_	Lap PPAN_E_
Sanctuary of Eukleia	5	3.175 ± 0.351	0.610 ± 0.103	3.010 ± 0.433	0.793 ± 0.201	0.006 ± 0.002	1.241 ± 0.371
Ancient Theatre of Mieza	5	3.673 ± 0.212	0.693 ± 0.030	3.303 ± 0.210	1.241 ± 0.147	0.006 ± 0.001	1.829 ± 0.235
Kasta Tumulus	5	0.868 ± 0.065	0.350 ± 0.030	0.673 ± 0.055	0.083 ± 0.018	0.001 ± 0.000	0.097 ± 0.025
Acropolis of Platania	5	3.122 ± 0.052	0.813 ± 0.073	2.913 ± 0.056	0.926 ± 0.052	0.006 ± 0.001	1.689 ± 0.067
Mosaic at Dion	4	1.374 ± 0.057	0.762 ± 0.110	1.158 ± 0.078	0.040 ± 0.007	0.009 ± 0.002	0.048 ± 0.004
Early Christian Basilica C	4	2.603 ± 0.376	0.824 ± 0.196	1.887 ± 0.315	0.568 ± 0.127	0.011 ± 0.005	0.435 ± 0.106
Olynthus	5	2.601 ± 0.283	1.333 ± 0.078	1.937 ± 0.238	0.412 ± 0.089	0.015 ± 0.002	0.416 ± 0.139

**Table 9:** Comparative fusion performance across all study sites (mean ± standard deviation). Spectral metrics were computed on the MS grid, while spatial metrics were derived from the NIR band of the native fused outputs. Spatial metrics are not reported for Brovey fusion where NIR injection is not applicable.

Method	Correlation	SAM (°)	Ten	Lap
PCA–PPAN_A_	0.84 ± 0.05	5.78 ± 0.62	2.49 ± 0.72	0.58 ± 0.32
PCA–PPAN_C_	0.95 ± 0.04	3.13 ± 0.48	0.77 ± 0.21	0.01 ± 0.01
PCA–PPAN_E_	0.93 ± 0.03	4.26 ± 0.57	2.13 ± 0.69	0.82 ± 0.41
Multiplicative–PPAN_A_	0.94 ± 0.03	2.70 ± 0.40	1.50 ± 0.40	0.28 ± 0.15
Brovey–PPAN_A_	0.82 ± 0.05	8.60 ± 1.10	N/A	N/A

## Data Availability

The datasets presented in this article are not readily available because they include imagery of archaeological sites and sensitive location-related information. Requests to access the datasets should be directed to the author.
